# The HCMV tegument protein UL88 degrades MyD88 and reduces innate immune activation

**DOI:** 10.1128/jvi.00414-25

**Published:** 2025-07-10

**Authors:** Rinki Kumar, Irene E. Reider, Madison Martin, Julia S. Simpson, Nicholas J. Buchkovich, Christopher C. Norbury

**Affiliations:** 1 Department of Cell and Biological Systems, The Pennsylvania State University College of Medicine 12310, Hershey, Pennsylvania, USA; Northwestern University Feinberg School of Medicine, Chicago, Illinois, USA

**Keywords:** HCMV, UL88, MyD88, immune activation

## Abstract

The human cytomegalovirus (HCMV) encodes the tegument protein UL88, which supports virus spread by mediating the degradation of the innate immune signaling adapter protein, myeloid differentiation primary response 88 (MyD88). MyD88 transduces signals in multiple innate immune pathways, including acting downstream of pattern recognition receptors and IL-1 cytokine family members. MyD88 is rapidly and robustly upregulated following exposure to HCMV, irrespective of viral gene expression and, even after infection, primarily within uninfected cells in a culture. However, UL88 was required to downregulate cellular MyD88 protein levels as HCMV spread through a culture. The N-terminal 181 amino acids of UL88 were required to associate with and downregulate MyD88 protein. MyD88 expression significantly suppressed virus spread by triggering the production of a heat-labile soluble factor. This factor was produced between ~3 and 6 days after initial infection and did not increase the expression of well-characterized interferon-stimulated genes (ISGs). Indeed, increased MyD88 expression downregulated the expression of almost all ISGs examined. UL88 overexpression suppressed IL-1β-induced NF-κB activation within a cell. UL88 also suppressed virus-induced translocation of NF-κB to the nucleus of uninfected neighboring cells in an infected monolayer. Furthermore, UL88 overexpression was required for effective HCMV spread following transfer of the virus from monocytes to a fibroblast monolayer. These data indicate that UL88 is a novel antagonist of the immune response that acts to enhance the natural spread of HCMV by targeting MyD88 and provides vital insight into the innate immune responses that can control HCMV spread.

**IMPORTANCE:**

The significant role of many viral genes encoded by HCMV that are not essential for replication in cell culture is often overlooked. Our study reveals the importance of UL88 for regulating the innate immune response by showing evidence for interaction with and downregulation of MyD88 protein. The UL88-dependent regulation of MyD88 is physiologically relevant, as infection is enhanced in the absence of MyD88, and spread from myeloid cells to fibroblasts is blunted in the absence of UL88. These results highlight yet another important interaction between HCMV and the immune system.

## INTRODUCTION

Human cytomegalovirus (HCMV) establishes a lifelong association with its host and is a major health concern. Infection, whether primary or after reactivation, is effectively controlled by the immune response and thus is often asymptomatic. However, HCMV poses a serious threat to immunocompromised individuals, in whom reactivation can lead to organ disease and mortality. Despite tremendous efforts that have been made to understand virus replication in the host, the functions of many HCMV genes remain elusive, likely because they are not required for replication in a cell culture monolayer. The proteins of the HCMV tegument have been associated with a number of functional roles, including transcriptional regulation of viral genes, downregulation of innate immune pathways, modulation of cell signaling pathways, and promotion of virion assembly. Many of the uncharacterized gene products of the tegument likely play a critical role in promoting infection in the more complex environment of the host.

Myeloid differentiation primary response protein 88 (MyD88) is a signaling adaptor that plays an important role in modulation of signaling after ligation of all TLRs except TLR3, and also after ligation of the IL-1 receptor family members IL-1R, IL-18R, and IL-1RL1 (ST2, the receptor for IL-33) (1–3). Signaling via MyD88 is required for effective antiviral immunity against many viruses, including hepatitis B virus (HBV) (4), dengue virus (5), KSHV (6), West Nile virus (WNV) (7), tick-borne encephalitis virus (8), and influenza virus (9). Previous studies have focused on understanding MyD88-dependent and -independent TLR signaling during HCMV infection (10–12) and the role of MyD88 in downstream induction of interferons via activation of NF-κB. Though informative, none of these studies have investigated the direct effect of MyD88 on virus spread and vice versa. Furthermore, no viral protein has previously been shown to directly target MyD88 during HCMV infection. Our data indicate that MyD88 is rapidly upregulated after exposure to HCMV, but that virus infection is not required for this upregulation.

Several studies have shown that HCMV encodes gene products that block host immune responses at multiple points (13). Viral proteins target the cytosolic nucleic acid sensor cGAS (14, 15) and signaling molecules downstream of nucleic acid sensors, including iRHOM, STING, MAVS, and STING/TBK1 (16). A cellular regulator of transcription downstream of innate immune signaling pathways, NF-κB, is targeted by many viral gene products, including IE86 (UL122) and UL26 (17–20). Viral pp65 and US9 target IRF3 to impact interferon-directed signaling (16, 21). However, HCMV modulation of signaling from cell surface and vesicular pattern recognition receptors, such as toll-like receptors (TLR), appears to be more nuanced. HCMV is reported to enhance TLR expression and signaling in macrophages (11), but may also target TLR expression via the viral proteins US7 and US8 (22) and viral miRNAs (23). The impact of this modulation of TLR signaling on HCMV replication and spread remains unclear.

We have previously studied the role of UL88, a tegument protein that exhibits late expression kinetics, in the assembly of HCMV. We showed that deletion of UL88 from the viral genome did not result in replication defects at either high or low multiplicity of infection (MOI) (24). UL88 does, however, have critical roles in tegument acquisition and alters the specific infectivity of the virus (24). In the absence of UL88, more genomes are required per infectious titer to infect at a comparable level to wild-type virus infection, indicating that more non-infectious particles are produced in the absence of UL88 (24).

Since the specific infectivity of HCMV was affected by the loss of UL88, we hypothesized that UL88 could play a role in modulation of host response to infection by altering the ratio of defective viral genomes in a virus population. However, we found that innate immune activation of fibroblasts, measured by enhanced expression of MyD88, was not altered in the absence of UL88, indicating that defective viral genomes or particles associated with the absence of UL88 do not play a role in immune evasion. However, as HCMV spread in a culture following low MOI infection, UL88 mediated degradation of MyD88 and prevented heightened activation of surrounding cells mediated by an HCMV-triggered soluble factor. In this way, UL88 facilitates HCMV spread.

## RESULTS

### MyD88 is upregulated in response to a low MOI HCMV infection and subsequently downregulated in a UL88-dependent manner as the virus spreads

**Fig 1:**
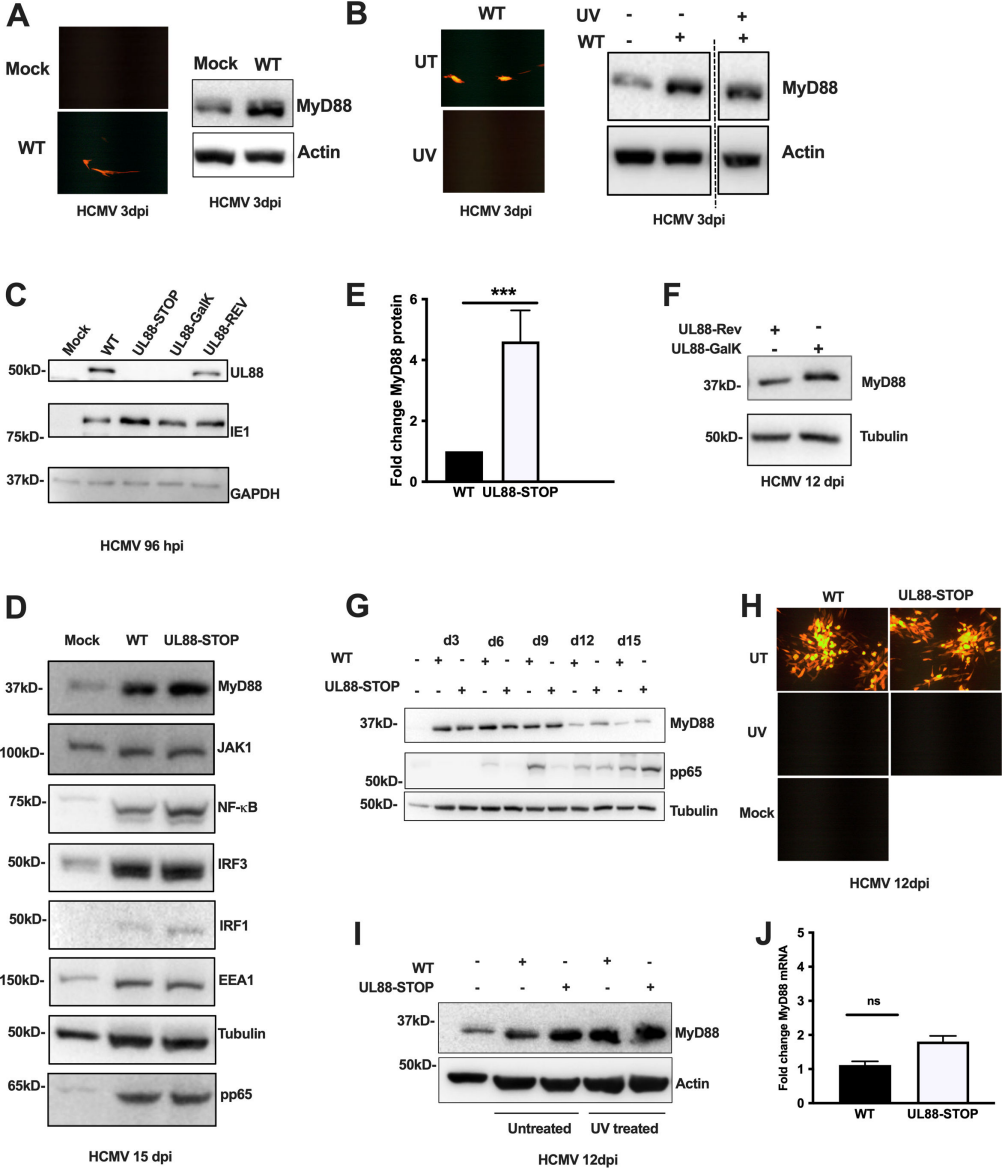
MyD88 levels during HCMV WT and UL88-STOP virus infection. (A) Protein lysates were harvested from fibroblasts either mock-infected or infected with TB40/E-mCh virus at an MOI of 0.05 at 3 dpi. Blots were probed for MyD88 and actin (loading control). Representative images of the infections show infected cells in red. (B) Protein lysates were harvested from fibroblasts either mock-infected or infected with untreated (UT) or UV-inactivated (UV) TB40/E-mCh or UL88-STOP-mCh virus at an MOI of 0.05 at 3 dpi. Blots were probed for MyD88 and actin (loading control). Representative images of the infections show infected cells in red. UT: untreated, UV: UV inactivated. (C) Protein lysates were harvested from fibroblasts infected with TB40/E WT, UL88-deficient (UL88-STOP and UL88-GalK), or UL88-Rev HCMV at an MOI of 3 at 96 hpi. Blots were probed for viral proteins UL88, IE1, and GAPDH as a loading control. (D) Protein lysates from fibroblasts, either mock-infected or infected with TB40/E WT or UL88-STOP HCMV at an MOI of 0.05, were harvested at 15 dpi. The lysates were analyzed by western blot (WB) and probed for MyD88, JAK1, NF-κB, IRF3, IRF1, EAA1, tubulin, and pp65 (viral protein). (E) The graph shows densitometry analysis from three independent experiments for each set at day 12 hpi. (F) Protein lysates were harvested from fibroblasts infected with either UL88-Rev or UL88-GalK virus at an MOI of 0.05 and harvested at d12 post-infection. Lysates were analyzed by western blot and probed for MyD88 and tubulin (loading control). (G) Protein lysates from fibroblasts, either mock-infected or infected with TB40/E WT or UL88-STOP TB40/E HCMV at an MOI of 0.05, were harvested every 3 days until 15 dpi. Blots were probed for MyD88, tubulin, and viral protein (pp65). (H) MRC-5 were either mock-infected or infected with untreated (UT) or UV-inactivated (UV) TB40/E-mCh or UL88-STOP-mCh virus at an MOI of 0.05. Representative images of the infections show infected cells in red at d12 post-infection. (I) Protein lysates from MRC-5 cells infected with TB40/E-mCh or UL88-STOP-mCh virus for 12 d (as pictured in H) were probed for MyD88 and actin (loading control). (J) RNA was harvested at 12 dpi from fibroblasts infected with TB40/E WT or UL88-STOP virus at an MOI of 0.05. MyD88 mRNA levels were analyzed by RT-qPCR and normalized to GAPDH. Fold changes are shown from three independent experiments. Results are shown as mean ± SD. *P < 0.05. Representative images of the infections show infected cells in red. UT: untreated, UV: UV inactivated.

To study the innate immune regulators of HCMV spread following an *in vitro* infection, we infected MRC-5 cells with an MOI of 0.05 and monitored the level of the innate immune signaling adapter MyD88 by western blot. 72 hours after infection with wild-type TB40/E HCMV (WT TB40/E), MyD88 was dramatically upregulated compared to its levels in cells that were mock-infected (Fig. 1A). As this upregulation occurred quickly after low MOI infection, when the majority of cells in the culture are not infected, we hypothesized that MyD88 upregulation may not require virus replication. To test this, we UV-treated the virus to a level that ablated expression of mCherry driven by the IE1 promoter (Fig. 1B) in our TB40/E mCherry strain (TB40/E-mCh), then examined MyD88 levels at 72 hours post-infection. We found that upregulation of MyD88 occurred similarly after exposure to either replication-competent HCMV or to UV-treated, replication-incompetent HCMV (Fig. 1B). Therefore, exposure of a culture of MRC-5 cells to HCMV at low MOI dramatically upregulates MyD88 in a manner that does not depend on virus replication or spread, and which likely occurs in uninfected, as well as infected, cells in the culture.

We have previously published that the HCMV tegument protein UL88 does not directly affect viral titers at high MOI but is required to incorporate some viral proteins into the tegument and to maintain the ratio of replicating vs non-replicating virions in a virus preparation (specific infectivity). As our findings left open questions regarding the utility of UL88 in HCMV infection, we subsequently sought to ascertain whether a UL88 deficiency was related to an ability to modulate the innate immune response. The UL88-deficient viruses (UL88-STOP and UL88-GalK), as well as the corresponding UL88 Revertant (UL88-Rev) virus used in this study, have been previously described and characterized (24). Crucially, we have shown that these viruses spread similarly following a low MOI infection with equivalent plaque-forming units, so any phenotype we observe is not due to differential infection with any of the viruses (24). Indeed, the data shown in Fig. 1B demonstrate that HCMV’s ability or inability to infect or spread in a culture does not alter the induction of MyD88. Figure 1C shows a western blot analysis of infected fibroblast lysates, which confirmed that the UL88-deficient viruses did not express UL88, while IE1 expression confirmed virus infection. UL88 expression was readily apparent in the wild-type infection and was restored after infection with UL88-Rev (Fig. 1C).

To investigate the potentially differential host responses to infection from our UL88-deficient viruses, we infected MRC-5 fibroblasts with WT TB40/E or UL88-STOP virus at an MOI of 0.05, then examined the protein levels of an array of innate immune signaling molecules including MyD88, IRF3, JAK1, STAT1, and IRF1, at day 15 post-infection. As above, we found an increase in MyD88 protein levels after infection with wild-type virus. Notably, we found that at d15 post-infection, MyD88 levels were higher in the samples infected with the UL88-STOP virus than with WT TB40/E (Fig. 1D). However, the levels of other innate immune signaling proteins, such as JAK1, STAT1, p-STAT1, IRF3, or IRF1, remained similar after infection with UL88-STOP or WT TB40/E (Fig. 1D). These results indicated a role for UL88 in the downregulation of virus-infection-induced MyD88. Across multiple experiments, we found that protein levels of MyD88 were on average ~4-fold higher after low MOI infection with a UL88-STOP virus vs a similar infection with WT TB40/E (Fig. 1D and E). To confirm that this effect is specific to UL88 and not a product of a bystander mutation in the UL88-STOP virus, we infected cells with UL88-GalK virus (another construct that does not express UL88), as well as with the UL88-Rev virus in which the expression of UL88 was restored (as in Fig. 1C). As shown in Fig. 1F, we observed a similar increase in MyD88 expression after infection with UL88-GalK relative to UL88-Rev at d12 post-infection, similar to infection with the UL88-STOP and WT viruses, respectively.

To investigate when during virus infection UL88 acts to downregulate HCMV-exposure-induced MyD88 expression, we measured MyD88 levels at 3-day intervals after infection. We found that MyD88 levels were constant from d3 until d9 post-infection, irrespective of whether the culture was infected with WT or UL88-STOP TB40/E (Fig. 1G). However, by d12, WT TB40/E had reduced MyD88 levels closer to those observed without infection than at d3 post-infection (Fig. 1G). Although levels of MyD88 12 days after infection with UL88-STOP virus were reduced from d9 levels with either WT TB40/E or UL88-STOP HCMV, they were higher than those in d12 WT TB40/E infection and remained so until at least d15 post-infection (Fig. 1G). The dramatic upregulation of MyD88 in the culture that did not depend upon virus replication and the subsequent downregulation of MyD88 at later time points implied that MyD88 was upregulated in both infected and uninfected cells, and that it was downregulated as HCMV spread through the culture. To test this hypothesis, we treated TB40/E-mCh or UL88-STOP virus expressing an mCherry tag (UL88-STOP-mCh) with UV as above and examined both the spread of virus and levels of MyD88 at d12 post-infection compared to infection with each untreated virus. Untreated TB40/E-mCh or UL88-STOP-mCh viruses both spread similarly in the culture by d12 post-infection, but UV treatment ablated discernible infection with either virus (Fig. 1H). As before, levels of MyD88 12 days after infection with WT TB40/E-mCh were still higher than levels in uninfected cells but were markedly lower than in cells infected with UL88-STOP-mCh HCMV (Fig. 1I). Levels of MyD88 in cells infected with UV-treated WT TB40/E-mCh or UL88-STOP-mCh HCMV were similar to those infected with replicating UL88-STOP HCMV (Fig. 1I). These results are consistent with the hypothesis that HCMV replication and spread are required for MyD88 downregulation after initial induction.

We also observed that the mRNA transcript levels of MyD88 and other proteins in the MyD88 signaling pathway (TIRAP, TRAM, TRIF, and TRAF6, not shown) are similar after low MOI infection with WT TB40/E or UL88-STOP virus (Fig. 1J). Thus, UL88 appears to modulate MyD88 at the protein level following a low MOI infection.

### UL88 is sufficient to downregulate MyD88

**Fig 2:**
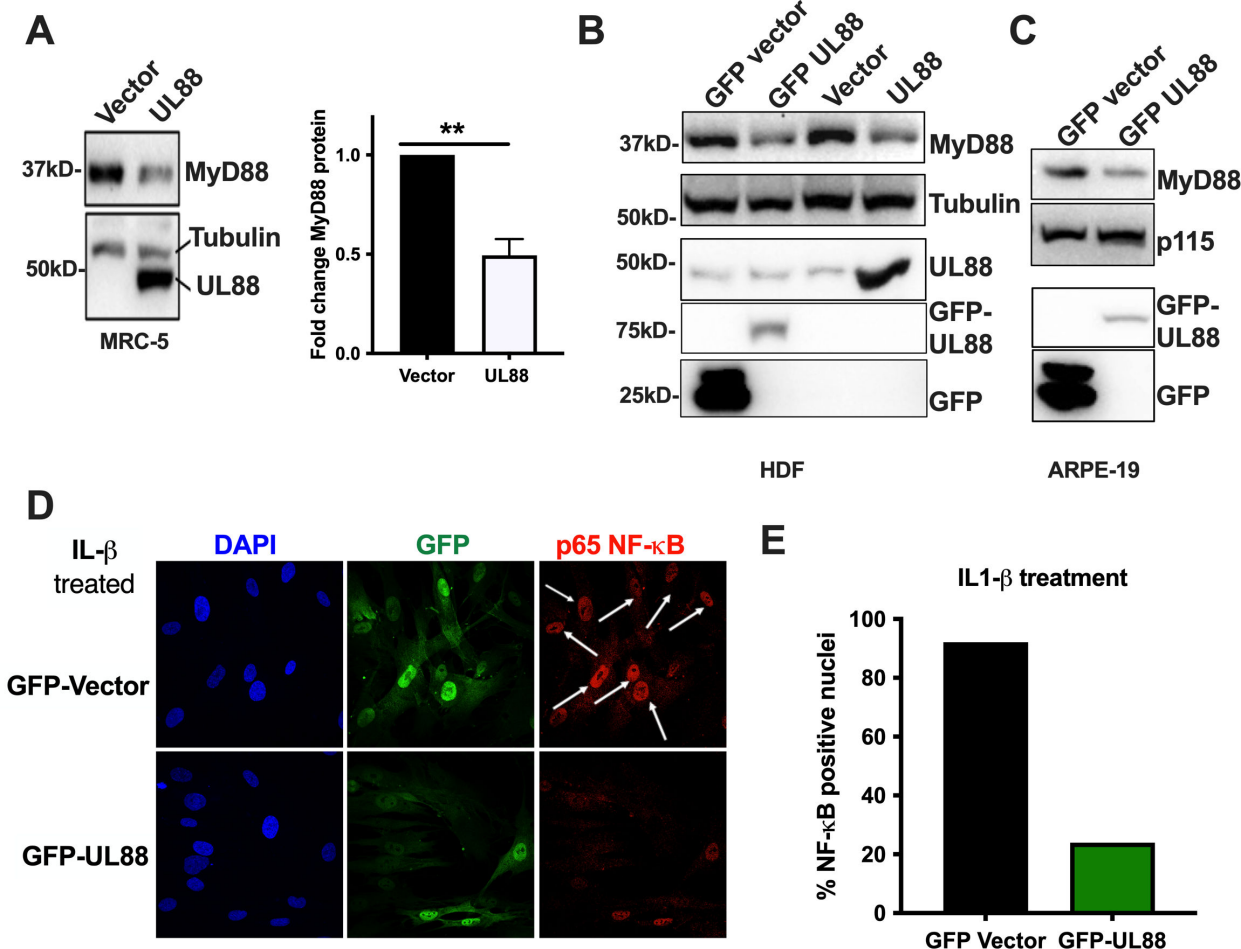
Effect of downregulating MyD88 on HCMV infection. (**A–C**) Fibroblasts (MRC-5 or HDF) and ARPE-19 were transduced with lentivirus expressing UL88 or vector and subjected to puromycin selection. Cells were harvested for either WB or RNA analysis. (A) MRC-5 cells expressing either vector or UL88 were analyzed by western blot and probed for MyD88, UL88, and tubulin. The graph shows MyD88 protein levels quantified by densitometry analysis from four independent experiments. Results are shown as mean ± SD. *P < 0.05. (B) Cell lysates from HDFs expressing GFP vector, GFP-UL88 vector, or UL88 were analyzed by western blot and probed for MyD88 and tubulin. (C) Cell lysates from ARPE-19 cells expressing GFP vector or GFP-UL88 were analyzed by western blot and probed for GFP, UL88, MyD88, and p115 (loading control). (D) Cells expressing either GFP-vector alone or GFP-UL88 were stimulated with IL-1β for 6 hours. Cells were fixed in PFA and stained for p65-NF-κB (red), GFP (green), and DAPI (blue). Cells were imaged on the confocal. (E) At least 10 different fields were imaged for each sample. The number of nuclei and NF-κB-positive nuclei was counted. The percentage of p65-NF-κB-positive nuclei is graphed.

Our data above indicate that UL88 can act in the context of an HCMV infection to reduce MyD88 levels. To examine whether UL88 is sufficient to downregulate MyD88 expression, we transduced MRC-5 fibroblasts (Fig. 2A), primary human dermal fibroblasts (HDF) (Fig. 2B), or ARPE-19 epithelial cells (Fig. 2C) with a lentiviral vector encoding either a native (Fig. 2A) or GFP-tagged version (Fig. 2B and C) of UL88, or with a control lentiviral vector encoding GFP. Untagged UL88 was probed using anti-UL88 antibody (~50 kd) while GFP-tagged UL88 and GFP were probed using the anti-GFP antibody (~75 kd and ~25 kd, respectively). The anti-UL88 and anti-GFP blots confirm the expression of UL88 in the respective samples. Importantly, irrespective of cell type, the expression of UL88 alone significantly downregulated the expression of endogenous MyD88 by at least twofold, indicating a broad effect that is not cell-type dependent (Fig. 2A through C).

We next sought to examine the functional consequences of UL88-mediated degradation of MyD88. The proximal result of MyD88-transduced signals is phosphorylation of NF-κB and its subsequent translocation into the nucleus, where a program of proinflammatory gene expression is triggered. To investigate the effects of UL88 expression upon NF-κB phosphorylation and nuclear translocation, we transduced fibroblasts with lentiviruses expressing UL88-GFP or GFP control, then stimulated the MyD88-dependent signaling pathway with IL-1β for 6 hours. As shown in Fig. 2D, IL-1β triggered nuclear localization of phospho-NF-κB in 90% of the cells expressing GFP alone (Fig. 2D and E). However, in UL88-GFP-expressing fibroblasts, in which we have shown that MyD88 levels are reduced (Fig. 2A through C), nuclear localization of phospho-NF-κB was significantly reduced (Fig. 2D and E). Therefore, UL88 expression alone suppressed MyD88-dependent IL-1β signaling.

### UL88 targets MyD88 for lysosomal degradation

**Fig 3:**
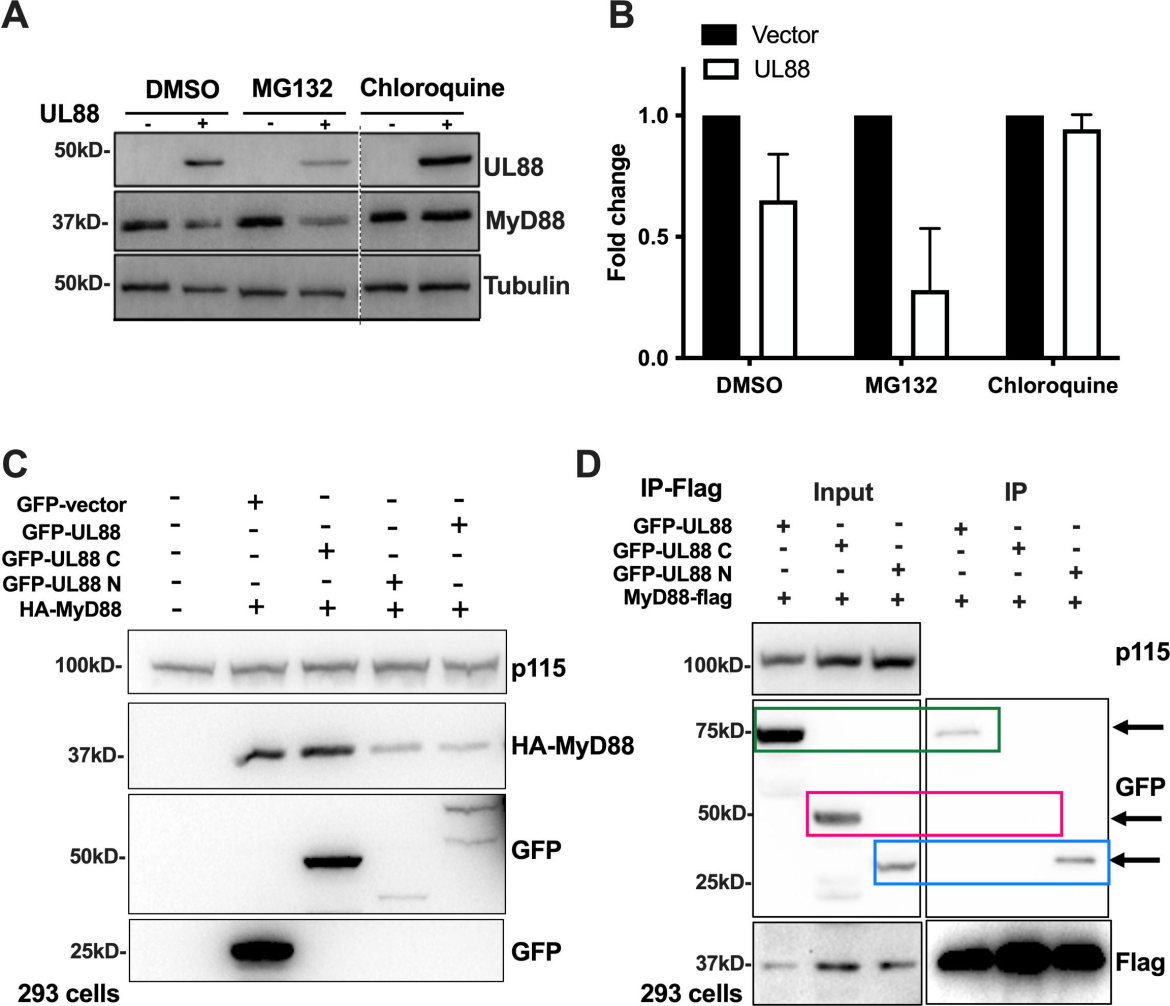
MyD88 is degraded by the N terminus of UL88 via the lysosomal pathway. (A) MRC-5 cells expressing either vector or UL88 were treated with DMSO, MG132 (10 µM), or Chloroquine (50 µM). Lysates were analyzed by western blotting. Blots were probed for UL88, MyD88, and p115 (loading control). Representative blots are shown. (B) Graph shows quantitation from three independent experiments as described in A. (C) 293 cells were co-transfected with HA-MyD88 and either GFP vector, GFP-UL88 full length, GFP-UL88 C, or GFP-UL88 N. Lysates were harvested 48 h post-transfection and analyzed by western blotting. Blots were probed for HA, GFP, and p115 (loading control). (D) 293 cells co-transfected with MyD88-flag and GFP-UL88 full length, GFP-UL88 C, or GFP-UL88 N. Lysates were harvested at 48 h post-transfection and subjected to co-immunoprecipitation using magnetic anti-flag beads. Samples were analyzed by western blot and probed for GFP, flag, and p115 as a loading control. Arrows point to GFP-UL88, GFP-UL88 N, and Flag-MyD88 in the IP blot.

As outlined above, UL88 is necessary (Fig. 1D through G) and sufficient (Fig. 2A through C) to downregulate MyD88 at the protein level. To investigate the mechanism responsible for the UL88-mediated downregulation of MyD88 protein, we examined whether the degradation of MyD88 mediated by transfected UL88 occurred in cytosolic or vesicular compartments. To accomplish this, we blocked cytosolic degradation using the proteasome inhibitor MG132 or blocked lysosomal degradation by preventing lysosomal acidification with chloroquine and examined MyD88 levels by western blot. In the presence of UL88 and inhibitor diluent (DMSO), levels of MyD88 were reduced, as they also were in the presence of the proteasome inhibitor MG132 (Fig. 3A). However, in the presence of chloroquine, levels of MyD88 were unchanged in the presence or absence of UL88 (Fig. 3A and B). These results likely indicate a vesicular route of degradation in an acid-sensitive compartment.

Next, we examined whether UL88 drove MyD88 degradation by associating with MyD88. We explored this possibility in two steps. First, we investigated the region of UL88 responsible for MyD88 degradation. Three constructs were generated: GFP-UL88, a GFP-tagged N-terminal region of UL88 (GFP-UL88-N, 1–180 aa), and a GFP-tagged C-terminal region of UL88 (GFP-UL88-C, 181–429 aa). Each construct was transfected into 293 cells in combination with HA-tagged MyD88. At 24 h post-transfection, lysates were harvested and subjected to western blotting. MyD88 levels were detected using an anti-HA antibody, and UL88 levels were detected using anti-GFP antibody. As seen in Fig. 3C, both the full-length GFP-UL88 and the N-terminal GFP-UL88 construct significantly reduced the levels of HA-MyD88, but the C-terminal GFP-UL88 construct did not. Therefore, the N-terminal region of UL88 is sufficient to mediate MyD88 degradation. Similar effects were observed with FLAG-tagged MyD88 (not shown). We then examined UL88/MyD88 associations by immunoprecipitation. To test for an association, either direct or indirect, between UL88 and MyD88, we co-transfected MyD88-FLAG and either GFP-tagged UL88, GFP-UL88-N (1–180 aa), or GFP-UL88-C (181–429 aa), and performed co-immunoprecipitation using anti-FLAG magnetic beads. A pulldown of MyD88-FLAG clearly also pulled down GFP-UL88 and GFP-UL88-N (green box and blue box, respectively), but not GFP-UL88-C (pink box) (Fig. 3D). This confirms that the N-terminus of UL88 associates directly or indirectly with MyD88 and likely drives its re-localization to vesicular compartments for degradation.

### MyD88 controls virus spread after low MOI infection

**Fig 4:**
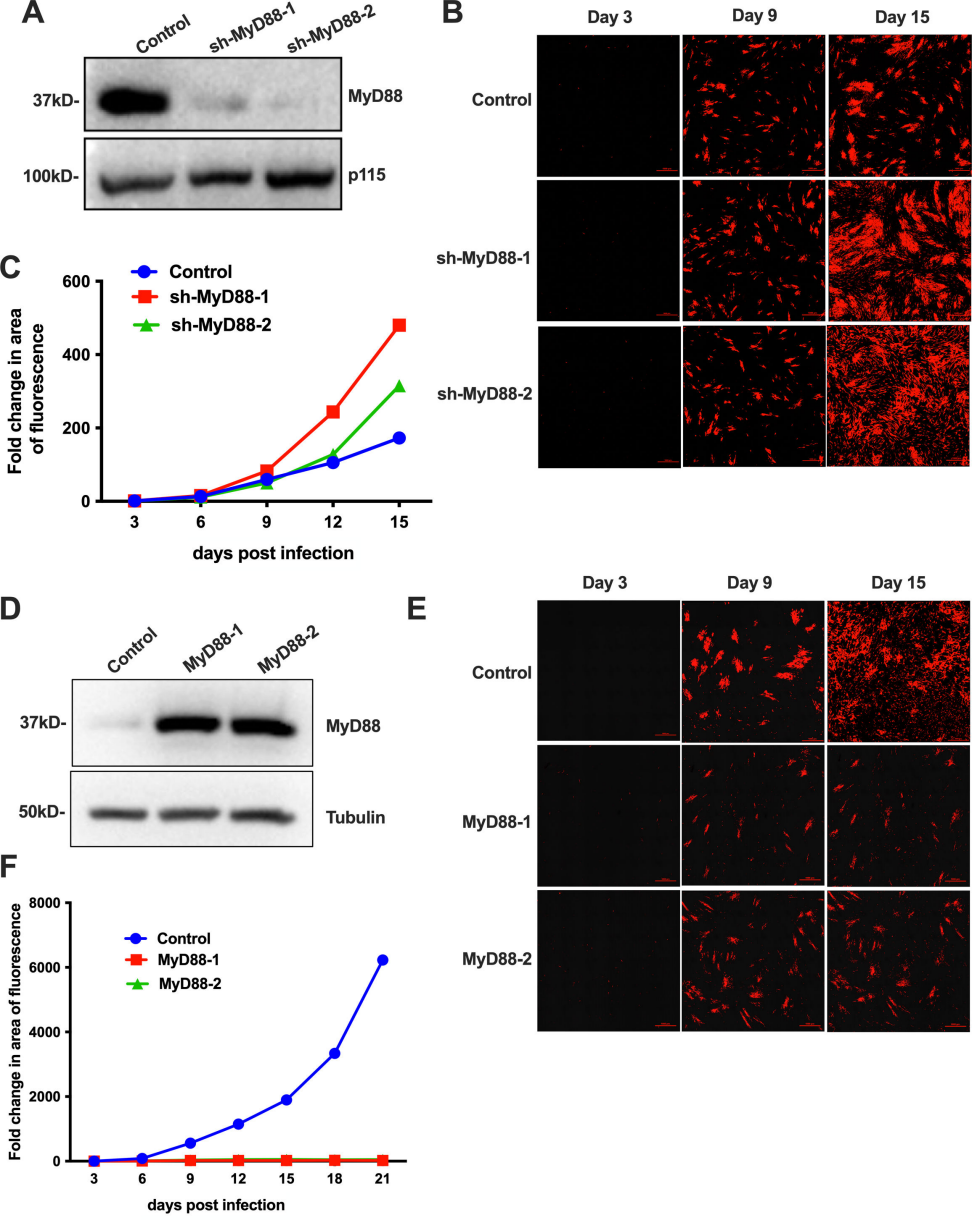
Lentivirus-mediated overexpression of MyD88 inhibits virus spread. (A) Lentivirus-mediated downregulation of MyD88. MRC-5 cells were transduced with control lentivirus or lentivirus encoding shRNA targeting MyD88 (shMyD88-1 and shMyD88-2) and selected using puromycin. Knockdown efficiency was assessed by western blot analysis. Blots were probed for MyD88 and p115 as loading controls. (B) The transduced and selected MRC-5 cells were infected with TB40/E-mCh virus at an MOI of 0.05, and the spread of the virus was monitored by confocal imaging. Representative images from days 3, 9, and 15 are shown. (C) The spread of infection was quantified as the area of fluorescence using the NIS Element software. (D) MRC-5 cells were transduced with lentivirus from control or two different cassettes encoding the MyD88 gene (MyD88-1 and MyD88-2) and subjected to puromycin selection. Cell lysates were harvested and run on a Western blot to analyze MyD88 levels. Tubulin served as a loading control. (E) The transduced and selected MRC-5 cells were infected with TB40/E-mCh virus at an MOI of 0.05, and the spread of the virus was monitored every 3 days by confocal imaging up to day 21. Representative images from days 3, 9, and 15 are shown. (F) The spread of infection was quantified as the area of fluorescence using the NIS Element software.

To directly examine the role of MyD88 in control of HCMV replication and spread *in vitro,* we transduced MRC-5 cells with lentiviral vectors expressing multiple shRNA targeting MyD88 from the Broad Institute TRC1.0 shRNA library. The successful knockdown of MyD88 with two individual shRNAs was confirmed by comparing MyD88 expression in the presence of these constructs to its expression in the presence of a control shRNA targeting GFP (Fig. 4A). We infected cell monolayers individually transduced with each of the three lentiviral vectors outlined above with WT TB40/E-mCh at an MOI of 0.05, then monitored the spread of the virus by microscopy. Representative images from days 3, 9, and 15 are shown for each in Fig. 4B. Quantitation of the area of fluorescence is shown in Fig. 4C. The virus spread was similar across all conditions up to and including d9 post-infection, but beyond that time point, was significantly higher in cells that had received shRNA targeting MyD88 (Fig. 4B and C). This indicates a role for MyD88 in the control of HCMV spread.

Having investigated the role of MyD88 by knocking it down, we used the opposite approach by transducing MRC-5 cells with either one of two lentiviruses expressing two distinct MyD88 constructs, or with a control lentivirus. The lentivirus-induced overexpression of MyD88 was confirmed by western blot (Fig. 4D). When we infected cells transduced with MyD88-expressing lentiviral vectors or a control vector, a striking difference was seen in HCMV spread (Fig. 4E and F). Figure 4E shows representative images from days 3, 9, and 15 for each sample. Figure 4F is a quantification of the area of fluorescence for each. Notably, differences between HCMV spread in cells transduced with vector or MyD88 were not evident until 9d post-infection, at which point the spread of HCMV in vector-transduced cells increased until d21; meanwhile, the viral spread in MyD88-transduced cells increased only very slightly after d9 (Fig. 4F). These results are consistent with a role for MyD88 in the control of infection.

### Increased MyD88 levels downregulate ISGs but reduce virus spread via a secreted factor

**Fig 5:**
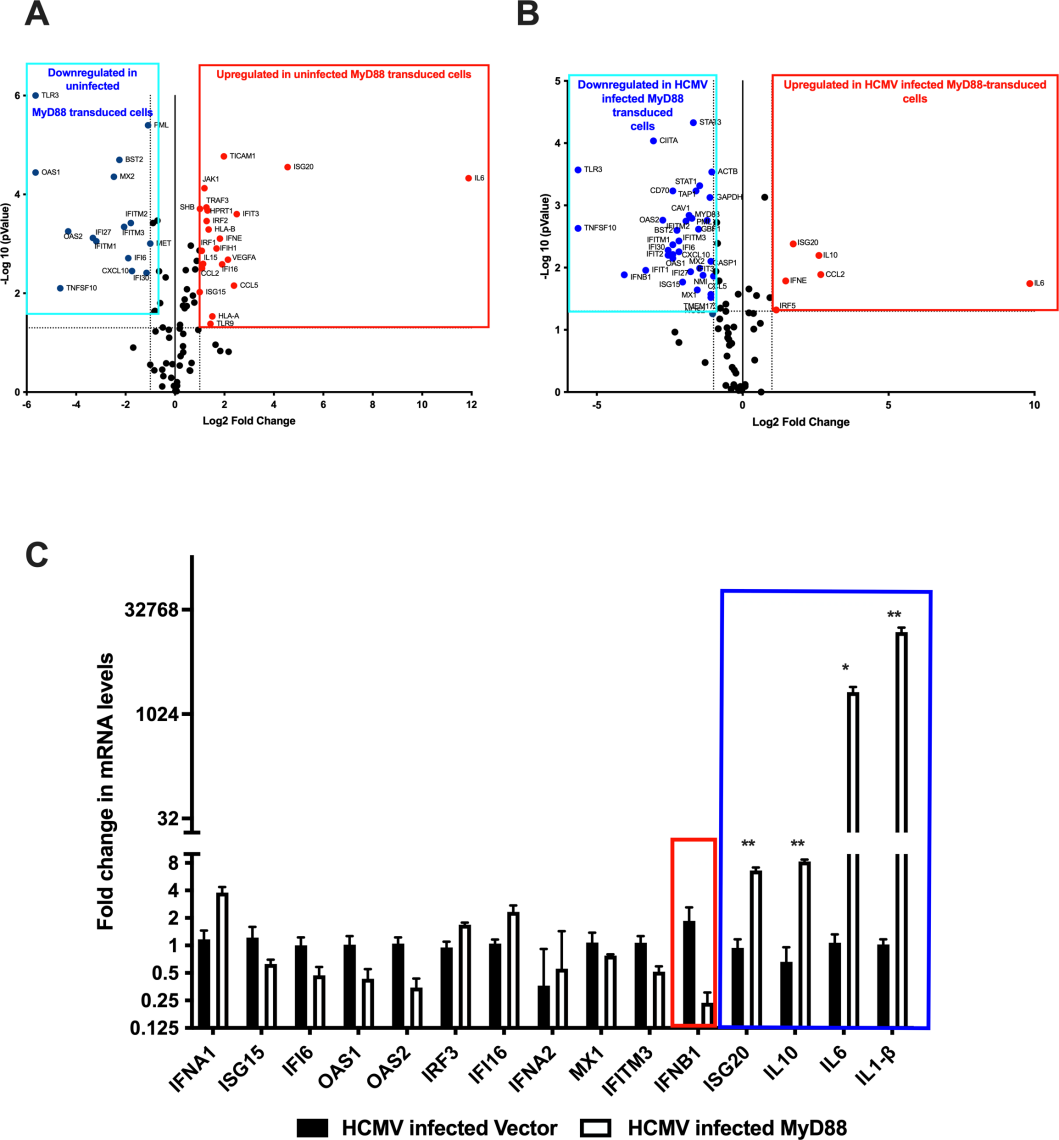
Effect of MyD88 overexpression on ISGs and spread of HCMV infection. (A, B) MRC-5 cells were transduced with lentivirus expressing a control or MyD88 gene and subjected to puromycin selection. Cells were either mock-infected or infected with TB40/E-mCh virus at an MOI of 0.05 and harvested at 7 dpi for RNA isolation, and samples were subjected to RT-qPCR analysis using the RT^2^ Profiler PCR array. (A) Volcano plot of the genes modulated in uninfected Myd88-transduced cells. The blue box represents genes that were downregulated, and the red box represents genes that were upregulated more than fourfold. (B) Volcano plot of the genes modulated in HCMV-infected MyD88-transduced cells. The blue box represents genes that were downregulated, and the red box represents genes that were upregulated more than fourfold. (C) The graph shows the representative ISGs that remained unchanged during HCMV infection in both conditions and ISGs that were significantly downregulated (red box) or upregulated by more than fourfold (blue box).

The reduction in HCMV spread in MRC-5 cells expressing high levels of MyD88 may be due to a strong induction of cytokines via MyD88 and, subsequently, the NF-κB pathway. However, there is significant crosstalk between genes induced by the NF-κB and those induced by the IFN-signaling pathways (25), many of which are antiviral in nature. Therefore, we hypothesized that antiviral interferon-stimulated genes (ISGs) could be induced by MyD88 expression in the context of HCMV infection, and we sought to identify these antiviral genes by profiling the expression of a set of 84 ISGs in cells transduced with a lentivirus expressing MyD88, or with a control lentivirus, and infected or mock-infected with HCMV. Contrary to our hypothesis, we found that MRC-5 cells transduced with MyD88-expressing lentivirus downregulated many antiviral ISGs including OAS1, MX2, IFI6, IFITM1, IFI27, and IFITM2, relative to MRC-5 cells transduced with control lentivirus (Fig. 5A). When these cells were infected with TB40/E-mCh at an MOI of 0.05 and harvested for RNA purification on d7 post-infection, a time point at which there were similar levels of virus present in cells transduced with MyD88 or the control lentivirus, this trend continued, but many additional ISGs were downregulated in the MRC-5 cells transduced with MyD88-expressing lentivirus relative to the MRC-5 cells transduced with the control lentivirus (Fig. 5B and C). Indeed, of the ISGs examined, only ISG20 was upregulated in MRC-5 cells transduced with MyD88-expressing lentivirus relative to the MRC-5 cells transduced with the control lentivirus (Fig. 5A), and this ISG20 expression was increased upon infection with HCMV (Fig. 5B and C). Therefore, it appeared unlikely that an intrinsic antiviral ISG was responsible for reducing the spread of HCMV in a monolayer of MRC-5 cells transduced with MyD88. Given these results, we then sought to examine the role of soluble factors in this process.

**Fig 6:**
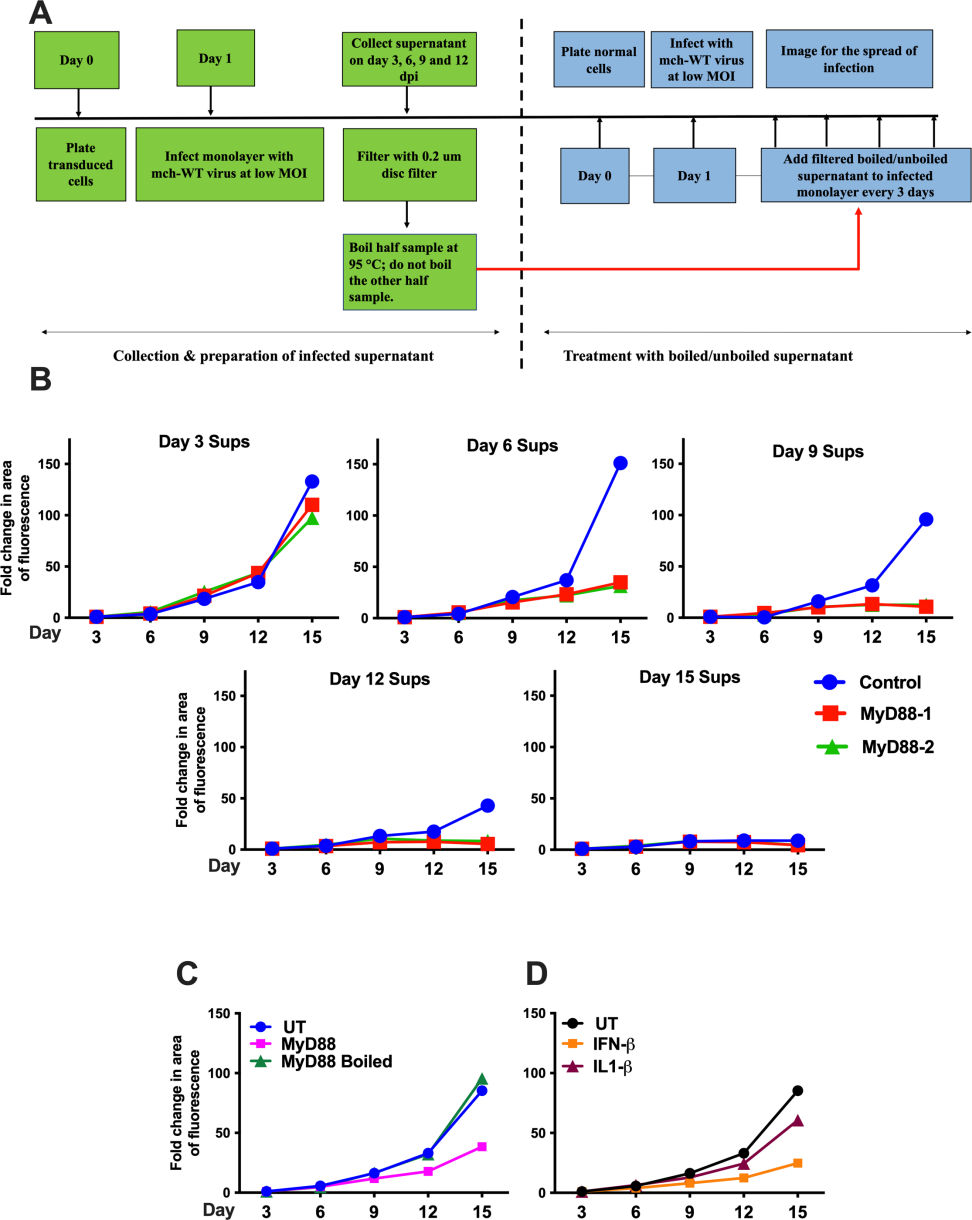
A MyD88-dependent heat-labile soluble factor reduces HCMV spread. (A) Schematic representation of the experiment methodology for this figure. (B) Supernatants from infected MRC-5 cells expressing either vector or MyD88 at an MOI of 0.05, with TB40/E-mCh, were collected every 3 days for 15 days and filtered through a 0.2 µm disc filter. The filtered supernatants from each day were then added to another monolayer of MRC-5 infected with TB40/E-mCh at an MOI of 0.05 every 3 days by removing the equivalent volume of the filtered supernatant from the well (therefore, 1:1 ratio with the media in the well). Cells were monitored for virus spread by imaging every 3 days. (C) Supernatants from infected MRC-5 cells expressing either vector or MyD88 were collected at 7 dpi and filtered through a 0.2 µm disc filter. They were then either untreated or denatured by boiling at 95°C before storage. The unboiled and boiled supernatants were added to another monolayer of MRC-5 infected with TB40/E-mCh at an MOI of 0.05 every 3 days by removing the equivalent volume of the filtered supernatant from the well (therefore a 1:1 ratio with the media in the well). The spread of the virus was monitored by imaging every 3 days. (D) A monolayer of MRC-5 infected with TB40/E-mCh at an MOI of 0.05 was treated with mock (UT), IFN-β (50 ng/mL), or IL1-β (100 ng/mL). The spread of the virus was monitored every 3 days by confocal imaging up to day 15. The spread of infection was quantified as the area of fluorescence using the NIS Element software. Results are shown as mean ± SD. *P < 0.05.

A number of transcripts encoding soluble factors, including IL-10, IL-6, and IL-1β, were upregulated in HCMV-infected cells transduced with MyD88 vs the control lentivirus (Fig. 5B and C). This raised the possibility that other biologically active soluble factors were being produced in HCMV-infected, MyD88-transduced cells, and that these factors were responsible for reducing HCMV spread. To examine the role of MyD88-induced soluble factors in modulating the spread of HCMV, we designed an experiment as laid out in Fig. 6A and described below. We performed a low MOI infection with TB40/E-mCh in MRC-5 cells stably expressing vector control or MyD88 and collected supernatants every 3 days until day 15. The spread was observed over a span of 15 days, with imaging and collection of supernatant every 3 days as shown in Fig. 6A. As we demonstrated above in Fig. 4B, differences in HCMV spread in vector versus MyD88-transduced cells were first observed between 6 and 9 days post-infection. Supernatants harvested on each third day were filtered using a 0.2 µm disc filter to remove any HCMV particles and stored prior to addition to HCMV-infected monolayers (as in Fig. 6A).

A fresh plate of MRC-5 cells was then infected at an MOI of 0.05. At day 3, when infection had been established, filtered supernatants from each third day (days 3, 6, 9, 12, and 15) of the infections described above were added to cells in a 1:1 ratio with existing media. Filtered supernatant was added every 3 days until day 15, and HCMV spread was imaged every 3 days as described above and shown in Fig. 6A. As shown in Fig. 6B, supernatant harvested from either control- or MyD88-transduced cells on d3 post-infection failed to curb HCMV spread in the fresh monolayer. Supernatants harvested from either vector control or MyD88-transduced cells at d12 or 15 post-infection both suppressed the spread of HCMV, indicating that infection of wild-type cells can produce soluble factors that inhibit HCMV spread, albeit at a time point at which spread has already occurred. However, supernatants harvested from MyD88-transduced cells on d6 and d9 effectively reduced HCMV spread in the fresh monolayer compared to supernatants from vector-transduced cells on the same days, with supernatant harvested on d9 proving most effective (Fig. 6B). Notably, even supernatant harvested on d9 did not discernibly reduce HCMV spread until d9 of the infection (d6 of treatment) (Fig. 6B). Therefore, the biologically relevant factor released from HCMV-infected MyD88-transduced cells is not released in sufficient quantity to be active until d6 post-infection, and this soluble factor requires an extended time period of exposure (>3 d) during an infection to be effective.

To confirm whether inhibition of HCMV spread by supernatant was mediated by a biologically active protein or lipid, we repeated the infection experiment in vector- and MyD88-transduced cells and collected supernatant at day 7 post-infection. The supernatant was filtered using a 0.2 µm disc filter and divided into two parts. One part was aliquoted and frozen immediately, and the other half was first boiled at 95°C to denature any soluble factors, then aliquoted and frozen. Figure 6C shows that boiled supernatant did not restrict virus spread, and in fact restored the ability of the virus to spread similar to levels observed with supernatant from vector-transduced HCMV-infected MRC-5 cells. Therefore, a MyD88-induced heat-labile factor is sufficient to reduce the spread of HCMV.

IL-1β mRNA is strongly induced following HCMV infection of MyD88-transduced cells (Fig. 5C), so we investigated whether IL-1β could be responsible for the blockade of HCMV spread by adding IL-1β to our monolayer of MRC-5 cells on d3 post-infection and every 3 days thereafter. As a positive control, we treated with IFN-β, which is known to inhibit HCMV spread, but which appears to be downregulated in MyD88-transduced HCMV-infected cells (Fig. 6D). The addition of IFN-β did mimic the action of supernatant from MyD88-transduced and HCMV-infected MRC-5 cells in reducing HCMV spread in a fresh monolayer, but IL-1β only marginally reduced HCMV spread in the fresh monolayer (Fig. 6D). Therefore, IL-1β is, at best, a minor player in the restriction of HCMV spread.

### UL88 expression attenuates acute inflammatory signaling

Having examined the effects of MyD88, the target of UL88, upon the inflammatory response and the resulting inhibition of HCMV spread, we then moved to directly examine the effects of virus-encoded UL88 upon innate immune activation after infection. As previously stated, the proximal event downstream from MyD88 signaling is phosphorylation and nuclear localization of NF-κB, which can then activate transcription of IL-1β, IL-6, and IL-10, all of which are upregulated in HCMV-infected cells expressing MyD88 (Fig. 5B and C). To examine the nuclear location of NF-κB following low MOI HCMV infection, we infected MRC-5 cells with mCherry-tagged wild-type (TB40/E-mCh), UL88-STOP (UL88-STOP-mCh), or UL88-Rev (UL88-Rev-mCh) HCMV at an MOI of 0.05. On d7 and d9 post-infection, we assessed nuclear localization of NF-ΚB in infected (red/mCherry^+^) or uninfected cells by co-staining with anti-p65 and DAPI.

**Fig 7:**
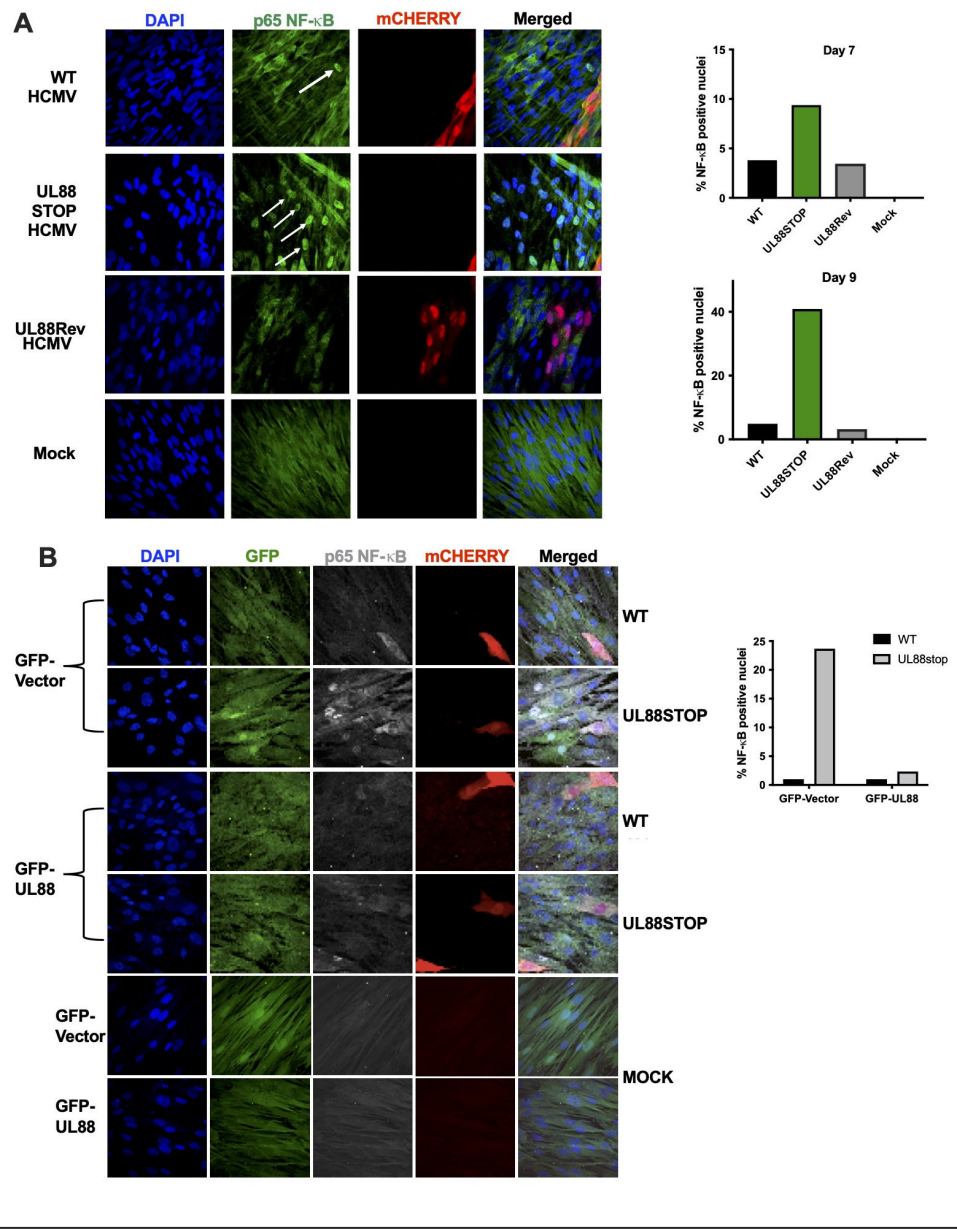
UL88 protein suppresses virus-induced NF-κB translocation. (A) MRC5 cells were plated on coverslips and infected with TB40/E-mCh, UL88-STOP-mCh, or UL88-Rev-mCh at an MOI of 0.05. Cells were fixed on day 7 and day 9 and subjected to IF analysis: DAPI (blue) and NF-κB (green), infection (red). NF-κB translocation into the nucleus was detected via staining, and cells with nuclear NF-κB signal were classified as “NF-κB-positive nuclei.” The percent of NF-κB-positive nuclei over total cells counted was calculated from at least 10 fields containing 100 cells each for each sample, as shown in the graph. (B) MRC5 cells expressing either GFP-vector or GFP-UL88 were plated on coverslips and infected with mCherry-tagged WT or UL88-STOP TB40/E virus at an MOI of 0.05. Cells were fixed on day 7 and day 9 and subjected to IF analysis: DAPI (blue), GFP (green), NF-κB (gray), and infection (red). NF-κB translocation into the nucleus was detected via staining, and cells with nuclear NF-κB signal were classified as “NF-κB-positive nuclei.” The percent of NF-κB-positive nuclei over total cells counted was calculated from at least 10 fields containing 100 cells each for each sample, as shown in the graph.

In mock-infected cells, there was some cytosolic NF-κB p65 staining, but no discernible nuclear localization of NF-κB p65 in any of the samples examined. After infection with TB40/E-mCh, there was obvious cytosolic NF-κB p65 staining, primarily in uninfected cells. However, there was very little discernible nuclear NF-κB p65 within mCherry^+^-infected cells, and very few (2%–3%) uninfected neighboring cells showed NF-κB staining in the nucleus (Fig. 7A). Similar to TB40/E-mCh, after infection with UL88-STOP-mCh HCMV, mCherry^+^ cells displayed little detectable nuclear NF-κB p65. However, in cultures infected with UL88-STOP-mCh, there were significant numbers of uninfected cells displaying nuclear localization of NF-κB p65 (~10% at day 7 and ~40% at d9, Fig. 7A). Infection with the UL88-Rev-mCh virus did not alter NF-κB p65 staining in infected cells and restored nuclear NF-κB staining in uninfected cells to the WT levels, indicating that this effect is specific to UL88 and not to any potential point mutants generated during the construction of the UL88-STOP-mCh virus. These observations are consistent with UL88-mediated modulation of innate immune activation within infected cells, an action that reduced activation of an antiviral state in neighboring uninfected cells.

To further establish that the effect we observed was mediated in a UL88-specific manner, we transduced fibroblasts with lentiviruses expressing either GFP or GFP-UL88, and then either mock-infected or infected with TB40/E-mCh or UL88-STOP-mCh virus. The monolayers were fixed and stained at day 7 and day 9. Figure 7B shows that the GFP-vector-expressing cells showed similar results to those in Fig. 7A, with uninfected cells demonstrating nuclear NF-κB staining after infection with UL88-STOP-mCh, but not wild-type, virus. However, in cells expressing GFP-UL88, NF-κB nuclear staining was reduced to the levels found in uninfected cultures, demonstrating that UL88 complementation could restore NF-κB activation.

### UL88 facilitates the transfer of virus from myeloid cells to fibroblasts via the production of proinflammatory cytokines, but not interferon-stimulated genes

**Fig 8:**
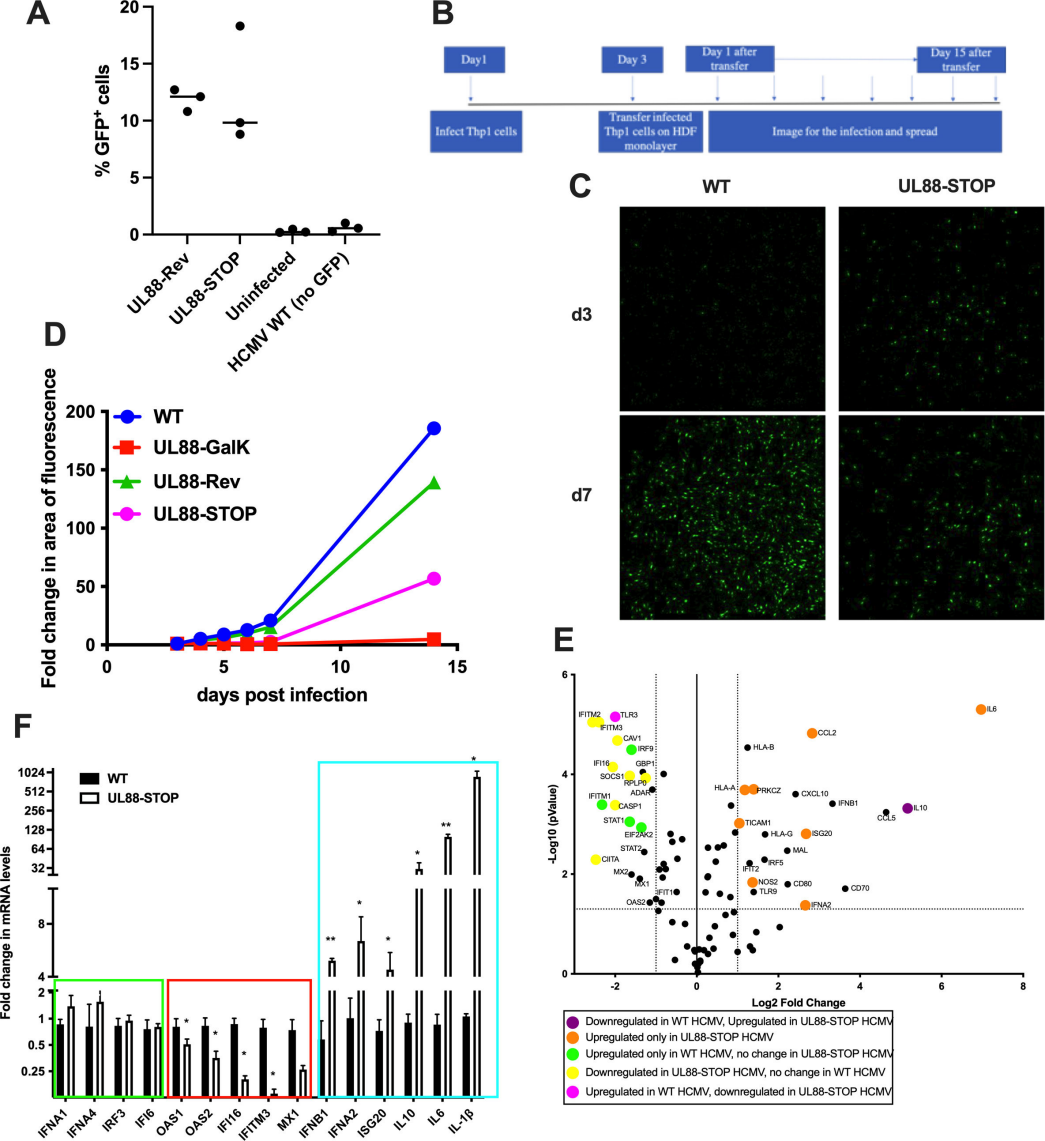
UL88 facilitates monocyte to fibroblast spread and downregulates HCMV-induced proinflammatory gene expression. (A) THP-1 cells were infected by spinoculation with an MOI of 20 with nonfluorescent TB40/E, UL88-STOP, or UL88-Rev, the latter two of which express GFP driven from the IE2 promoter or were left uninfected. GFP fluorescence was measured by flow cytometry at d5 post-infection. (B) Schematic representation of the experiment methodology for C and D. At day 3, 2.5 × 10^5^ THP-1, of which ~ 2.5 x 10^4^ were HCMV-infected, were transferred onto a monolayer of ~2.5 x 10^5^ HDFs, and virus spread was monitored every 3 days by confocal imaging up to day 15. (C) Representative images of TB40/E-GFP and UL88-STOP from day 3 and day 7 are shown. (D) The spread of infection was quantified as the area of fluorescence using the NIS Element software. The graph shows fold change in area of fluorescence for each virus. (E) MRC-5 cells were infected with TB40/E-mCh or UL88-STOP-mCh virus at an MOI of 0.05. Cells were harvested for RNA isolation at 7 dpi. Antiviral gene expression was profiled using a set of 84 ISGs by qPCR. Results are plotted as a volcano plot depicting the modulated genes compared between TB40/E WT HCMV and UL88-STOP HCMV infection. (F) ISGs known to be modulated in HCMV infection are shown with their relative fold change in mRNA levels (normalized to GAPDH) between TB40/E WT and UL88-STOP virus infection. The graph shows the genes that remained unchanged (green box), downregulated (red box), or upregulated (blue box) in TB40/E WT vs UL88-STOP HCMV-infected cells. Results are from biological triplicates.

Although we have previously described that UL88 is not required for efficient spread of HCMV in a fibroblast monolayer (24), our data demonstrating a significant role for both UL88 and MyD88 in innate immune activation indicated that UL88 may play a vital role in HCMV pathogenesis. A key step within the HCMV life cycle *in vivo* is the spread from cells of the myeloid lineage, in which HCMV establishes latency, to neighboring tissue. This transfer of virus is a key step in viral dissemination and HCMV-mediated disease. Using a co-culture system, we tested whether UL88 is required for the transfer of infectious virus from myeloid THP-1 cells to HDF fibroblasts. We infected THP-1 cells with nonfluorescent TB40/E HCMV, UL88-STOP, or UL88-GalK viruses (neither of which expresses UL88), or with UL88-Rev virus. Each virus, aside from the nonfluorescent wild type, encodes a GFP reporter, dependent upon expression of the major immediate early proteins, to allow visualization of virus spread. THP-1 cells were infected at an MOI of 20 by spinoculation, and by d5 post-infection, it was clear that UL88 expression did not alter infection, and approximately 10% of the cells were expressing immediate early proteins as measured by surrogate GFP expression (Fig. 8A). At day 3, all THP-1 cells (of which ~10% were infected) were transferred onto a monolayer of HDFs at a ratio of 1 infected THP-1 cell to ~12 uninfected HDFs. A schematic of the experimental protocol is shown in Fig. 8B. Virus spread from the THP-1 cells (in which further spread did not occur in a monoculture) to HDFs was monitored by imaging daily until day 7 and every 3 days thereafter until day 21 (Fig. 8B). Representative images either 3 or 7 days after infection with TB40/E wild-type or UL88-STOP HCMV are shown in Fig. 8C. By day 7 post-infection, UL88-STOP HCMV had exhibited markedly less spread to the fibroblast layer of cells than wild-type TB40/E (Fig. 8C). Quantification of HCMV spread from THP-1 cells to the fibroblast monolayer shows that the wild-type TB40/E or UL88-Rev viruses spread in the monolayer, but that the spread of the UL88-STOP or UL88-GalK HCMV was impaired (Fig. 8D). Therefore, UL88 is required for efficient spread of HCMV from cells of the myeloid lineage to neighboring cells.

We have demonstrated above that UL88 expression inhibits NF-κB nuclear localization in uninfected cells within an HCMV-infected culture. The NF-κB pathway can induce proinflammatory cytokines, as described above, but can also induce antiviral ISGs that could readily account for the reduced myeloid-fibroblast spread observed in the absence of UL88. To examine the potential role of ISGs in this type of viral spread, we profiled the expression of a set of 84 ISGs in MRC-5 cells infected with TB40/E-mCh or UL88-STOP mCh HCMV at an MOI of 0.05 for 7 days. As expected, transcripts of genes encoding a number of soluble cytokines downstream of the NF-κB pathway, including IL-10, IL-6, and IL-1β (Fig. 8E and F), were induced more strongly in MRC-5 cells infected with UL88-STOP-mCh HCMV than with TB40/E-mCh. However, as with infection of MyD88-expressing cells, we observed that the UL88-STOP-mCh HCMV infection, in which MyD88 levels are higher, actually downregulated a number of antiviral ISGs, including IFITM1, IFITM2, IFITM3, MX1, and IFI16 (the nuclear sensor that plays a role in sensing of HCMV) (Fig. 8E and F). Indeed, only one antiviral ISG, the RNA exonuclease ISG20, was upregulated in MRC-5 cells infected with UL88-STOP-mCh HCMV (Fig. 8E and F). In the context of UL88-STOP-mCh HCMV infection, we also observed upregulation of the Type I interferons IFN-β and IFN-α2 (Fig. 8E and F), although IFNα1, IFNα4, and IRF3 were similarly expressed after infection with TB40/E-mCh or UL88-STOP-mCh HCMV (Fig. 8E and F). Therefore, we observed an upregulation of both interferon and the inflammatory products of the NF-κB pathway, but contrarily, decreased transcripts of antiviral ISGs after infection with UL88-deficient HCMV.

## DISCUSSION

Dissemination of HCMV through a host requires infection of myeloid cells, followed by spread from those cells to other target cell types. Each of these instances represents a potential inflection site at which the host immune system can reduce or slow infection. The lack of tractable small animal models for HCMV does not typically allow realistic modeling of immune evasion strategies in a natural host. However, by studying low MOI infection as well as the spread of virus from infected myeloid cells to fibroblasts, we have been able to identify and characterize a previously unappreciated role of the HCMV tegument protein UL88 in modulation of the innate immune response in infected cultures.

Upon exposure of a fibroblast monolayer to a low MOI of HCMV, there was a rapid and dramatic upregulation of the innate immune signaling adapter MyD88. MyD88 transduces signals from the majority of cell surface and endosomal TLRs, with the exception of TLR3 and some signaling through TLR4. MyD88 is also involved in the transduction of signals from IL-1R family members, including IL-1R, IL-18R, and IL-33R (ST2). MyD88 upregulation occurred even when UV-treated HCMV was used, so viral gene transcription was not required for the induction of this signaling adapter. Indeed, our visualization of cellular activation through a MyD88-dependent signaling pathway, via nuclear localization of phosphorylated NF-κB, occurred almost exclusively in cells in the culture that were not infected with HCMV. This implied that MyD88 upregulation occurred in response to the mere presence of HCMV virions or other materials in the HCMV viral prep. Our virus preps were ultracentrifuged through a 20% sorbitol cushion to remove cellular debris, but it is notoriously difficult to remove all traces of ligands for pattern recognition receptors from a virus preparation. As a result, it is unclear whether MyD88 expression was upregulated in response to virions or cellular debris.

We have previously described a role for UL88 in maintaining the specific infectivity of virions, as its absence leads to altered incorporation of other tegument proteins such as UL47, UL48, and pp71 (24). However, a screen of signaling molecules involved in innate immune signaling revealed that UL88 was required to downregulate MyD88. This suggested a dual function for UL88 in both maintaining virus structural integrity and facilitating modulation of host immune response. It is possible that these two functions are linked, possibly via UL88’s role in mediating efficient incorporation of the tegument proteins pp71, UL47, and UL48 into mature virions, which could result in the presence of higher proportions of defective interfering particles or genome-containing virions in virus preps of HCMV lacking UL88. We have previously shown that an increased number of virions can enter cells following exposure to UL88-deficient HCMV, consistent with this possibility (24). However, UV inactivation of WT or UL88-deficient HCMV did not affect the ability of virus preparations to cause an increase in MyD88 expression. By contrast, UV-inactivation of HCMV did inhibit the ability of HCMV to downregulate MyD88. This is consistent with a role for UL88 in HCMV spread to MyD88-expressing cells during a low MOI infection and is inconsistent with a role for UL88 production of defective viral particles or genomes (which would be unaffected by UV treatment) in modulation of MyD88 expression.

UL88 alone was sufficient to downregulate MyD88 in the absence of virus infection and was required for this downregulation in the context of infection. Therefore, UL88 appeared to be acting alone or with cellular cofactors to accomplish the task of immune modulation. The downregulation of MyD88 did not occur at the transcript level but rather was a result of targeted protein degradation. The N terminus of UL88 interacted, directly or indirectly, with MyD88 and was sufficient to downregulate MyD88 protein. Previous studies have identified E3 ligases that promote the polyubiquitination and proteasomal degradation of cytosolic MyD88 (26–28). However, we found that downregulation of MyD88 was not prevented by inhibitors of the proteasome, but rather was blocked by chloroquine, which blocks endosomal acidification. Therefore, we hypothesized that autophagic uptake of MyD88 into vesicular compartments led to its subsequent degradation. However, MyD88 did not co-localize with a classical marker of autophagosomes, p62, and degradation did not require the selective macroautophagy/autophagy receptor TAX1BP1 (not shown). Therefore, UL88 may target MyD88 for degradation by binding to a membrane protein that is targeted to lysosomes via the ESCRT pathway; alternatively, non-classical autophagy and processes such as a mitophagy-like pathway may also be involved.

A role for MyD88 in the modulation of HCMV spread *in vitro* has not been previously described. We demonstrated that the functional consequence of UL88 expression was a blockade of signaling through the MyD88-dependent IL-1R pathway, indicating that signaling through this and potentially other MyD88-transduced signaling pathways is likely blocked in HCMV-infected cells. Since cells of the myeloid lineage have been shown to robustly activate immune signaling pathways following HCMV infection (29–31), UL88 may be important to counteract this immune signaling, and this could explain why UL88 alters myeloid-to-fibroblast spread, but not spread within a fibroblast monolayer. We appreciate that the model of spread of HCMV from the THP-1 myelomonocytic cell to a monolayer of fibroblasts is not an ideal system, and that some studies have indicated the THP-1 cells do not support HCMV replication with a blockade in IE1/2 expression (32). However, we did observe GFP driven by the IE2 promoter as a surrogate of infection in ~10% of THP-1 cells, cells which we then added to a monolayer of HDF in a ratio of 1 infected THP-1 to ~12 HDF. The spread we observed from THP-1 to HDF could be a result of replicating virus, virus that has been internalized by THP-1 and then released, or virus particles that have been held on the surface of THP-1 and then released to allow infection of HDF. Nonetheless, the presence of THP-1 in the culture renders efficient virus spread dependent upon UL88 expression. The effects of UL88 were apparent only when spread was examined, indicating that the absence of UL88 expression did not alter infection. Indeed, the effects of UL88 were only apparent following a low MOI spread infection, likely because of the rapid and robust upregulation of MyD88 following exposure to a virus prep vs. the late expression kinetics of the tegument protein UL88. In addition, the functional effects of UL88 in preventing spread likely stem from inhibition of the MyD88-mediated production of a soluble mediator(s) that can confer greater resistance against virus spread to neighboring cells, a phenotype that is functionally irrelevant in a high MOI infection.

Indeed, the spread of UL88-deficient virus from an infected myeloid cell population to fibroblasts, or of wild-type virus in MyD88-transduced fibroblasts, was characterized by an activation of innate immune signaling in uninfected cells in the culture. Our data indicated that MyD88-transduced cells released soluble cytokines that imparted an antiviral state on neighboring uninfected cells, slowing the spread of HCMV in the culture. Similar gene patterns were induced in HCMV-infected MyD88-transduced cells and cells infected with UL88-deficient HCMV, so it is likely that the same cytokines act upon uninfected cells in the culture in each case. At this time, the soluble mediators that confer resistance to HCMV spread remain uncharacterized but likely have a greater effect when THP-1 cells are in the culture, perhaps due to enhanced effects upon these cells. If the induction of soluble mediators that alter HCMV spread is MyD88-dependent, the induction likely lies downstream of the NF-κB pathway. The classical transcripts induced by NF-κB include IL-1, IL-6, and TNFα, and we observed induction of each of these, along with IL-10, in both HCMV-infected MyD88-overexpressing cells and cells infected with UL88-deficient HCMV. However, both IL-10 and IL-6 are typically advantageous for the growth of HCMV or other herpesviruses, and inflammation may facilitate HCMV replication and dissemination (33). Nonetheless, it is clear that the inhibition of HCMV spread in the presence of lentivirus-expressed MyD88 was dependent upon an extrinsic factor that was not produced in significant quantities until day 6 after infection. IL-1β can induce antiviral ISGs in fibroblasts to control virus replication (34), and HCMV deploys a number of strategies to inhibit the production and action of IL-1β (35). However, although IL-1β transcripts were induced by d7 post-infection, IL-1β treatment of fibroblasts did not reproduce the strong inhibition of HCMV spread we observed in MyD88-expressing cells. Therefore, classical NF-κB-induced proinflammatory transcripts may not be responsible for the effect of UL88 modulation of MyD88 signaling upon HCMV spread.

In addition to classical NF-κB-induced proinflammatory transcripts, there is significant crosstalk between the NF-κB signaling pathway and the interferon (IFN) signaling pathways, which induce antiviral ISGs (25). Previous studies have focused on understanding the MyD88-dependent and -independent TLR signaling during HCMV infection and on MyD88’s role in inducing downstream induction of IFNs (10–12). We did find that induction of both IFN-β and IFNα2 transcripts was increased after infection with UL88-deficient HCMV, indicating that Type I IFNs could be the exogenous factor that slows HCMV spread in MyD88-transduced cultures. Indeed, the addition of IFN-β to these cultures could partially reproduce the effect of supernatant from HCMV-infected cultures on HCMV spread in fresh cultures. However, the majority of antiviral ISGs, including OAS1, MX2, IFI6, IFITM1, IFI27, and IFTM2, were downregulated in cells transduced with MyD88 (in the presence or absence of infection) and were also downregulated in cells infected with UL88-deficient HCMV vs wild-type HCMV. Several ISGs—including IFIT1, IFIT2, IFIT3, MX1, Mx2, CXCL10, and ISG15—are typically upregulated transcriptionally following HCMV infection, independently of type I IFN-initiated JAK-STAT signaling (36), but this process appears, somewhat surprisingly, to be perturbed in the presence of increased levels of MyD88.

The only antiviral ISG in our screen that we found to be upregulated in the presence of increased levels of MyD88 and HCMV infection was ISG20. ISG20 is an RNA exonuclease that is strongly induced by Type I IFNs and which may also have a physiological impact on cellular functions in the absence of ongoing IFN responses (37, 38). When overexpressed *in vitro*, ISG20 restricted infections by EMCV, vesicular stomatitis virus, influenza virus, HIV and Sindbis virus, WNV, and HCV (39–42). Knowledge of the activity of ISG20 against DNA viruses is restricted to reports of Hepatitis B virus replication (43), although notably, HCMV pp65 is known to inhibit ISG20 induction (21). Therefore, although ISG20 may play a role that requires further investigation, it is equally possible that any effect of Type I IFN-induced ISGs downstream of MyD88-mediated signaling on the spread of HCMV may be mediated by ISGs that are not in our screen.

In summary, HCMV-expressed UL88 targets MyD88 within infected cells for degradation in lysosomal compartments. This impairs production of soluble mediators, likely downstream of the NF-κB pathway, which are normally produced from infected cells and which trigger in neighboring cells an antiviral state that reduces virus spread. Extensive further studies will be required to identify the mechanism that mediates resistance to virus spread in the absence of UL88—but once identified, this mechanism could potentially be manipulated in clinical settings to reduce virus spread in immunocompromised individuals.

## MATERIALS AND METHODS

### Cell culture

Normal human dermal fibroblasts (HDFs) (catalog no. 106-05n; Cell Applications Inc.), MRC-5 cells (ATCC CCL-171), HEK 293, and HEK 293TN were maintained in Dulbecco’s modified Eagle’s medium (DMEM) (Corning) containing 10% fetal bovine serum, 2 mM GlutaMAX (Gibco), 100 U/mL penicillin, and 100 µg/mL streptomycin (Corning). Cells were maintained at 37°C under 5% CO_2_. ARPE-19 cells (ATCC CRL-2302) were cultured in DMEM F12 media containing 10% fetal bovine serum, 2 mM GlutaMAX (Gibco), 100 U/mL penicillin, and 100 µg/mL streptomycin (Corning). The human monocyte cell line, THP-1 (ATCC, TIB-202) was maintained in RPMI (Corning) supplemented with 10% fetal bovine serum (HyClone), 2 mM GlutaMAX (Gibco), 100 U/mL penicillin, 100 µg/mL streptomycin (Corning), and 1.75 µM ß-mercaptoethanol.

### Plasmids and cloning

The pEGFPC1-1-UL88 and deletion constructs of UL88 N (N-terminal: 1–180 aa) and UL88 C (C-terminal: 181–429 aa) in the pEGFP-C1 vector backbone were generated as described previously (24). GFP versions were also cloned using the pEGFP-C1 vector backbone. An HA-tagged construct of MyD88 was purchased from Addgene (12287). MyD88 was then amplified and cloned into different vector backbones like pCDH, pCDH-2AGFP, pCDNA3.1 with the FLAG tag, and pEGFPC1 using XhoI/BamHI restriction sites. All plasmid sequences were verified by Sanger sequencing (Genewiz).

### Lentivirus preparation and transduction

Lentivirus was generated using the third-generation packaging system: pCMV-VSV-G (gift from Bob Weinberg, Addgene #8454), pMDLg-RRE (gift from Dr. Didier Trono, Addgene #12251), and pRSV-Rev (gift from Dr. Didier Trono, Addgene #12253) in 293TN cells as described previously (24). The MyD88 (TRCN0000008024 (clone #1), TRCN0000008026 (clone #2)) shRNA plasmids were from the TRC1.0 shRNA library from Broadway Institute, obtained through the Genome Sciences Core at PSU. Scrambled control was a gift from Dr. David Sabatini (Addgene #1864). The puro sequence was replaced with a puro-2A-EGFP sequence as described previously (44). For lentivirus transduction, sub-confluent fibroblasts were seeded overnight and transduced with lentivirus and 8 µg/mL Polybrene (Sigma-Aldrich). Transduced cells were selected using 2 µg/mL puromycin (Thermo Fisher).

### HCMV infections, growth curves, and virus titrations

WT TB40/E, TB40/E expressing mCherry (TB40/E-mCh), TB40/E expressing EGFP (TB40/E-EGFP), and UL88-STOP, UL88-GalK, and UL88-Rev viruses were generated as described and characterized previously (1, 44). To generate virus stocks, purified BAC DNA was electroporated into MRC-5 cells as described previously (1). Briefly, infected cells were used to infect MRC-5 cells in roller bottles (Greiner) to produce larger stocks of virus for experimental infections. Virions produced in roller bottles were concentrated by ultracentrifugation on a 20% sorbitol cushion at 20,000 rpm for 1 hour at 20°C in a Beckman SW32 rotor. HCMV infections were carried out at an MOI of 0.05 for multistep growth curves (low MOI) and at an MOI of 3 for single-step growth curves (high MOI). Virus-infected cells were incubated for three hours at 37°C before the cells were washed twice with 1× PBS, and fresh media was added. Where applicable, the virus was UV inactivated under a Mineralight ultraviolet lamp (shortwave UV at 254  nm) for 30 min at a distance of 10 cm.

For viral titer assays, samples were harvested at the time points indicated post-infection for each experiment by scraping the cells into the medium, sonicating 10 times with 1 second pulses, vortexing for 15 seconds, and centrifuging at 13,000 rpm for 10 min. Supernatants were collected and flash-frozen in liquid nitrogen before storage at −80°C. For western blot analysis or RNA isolation, samples were harvested at the indicated time point and processed as described below.

HCMV stocks and samples for growth curves were titrated by serial dilutions on MRC-5 cells and were quantified by the immunological detection of immediate early proteins as described previously (1). The IE-1 mouse antibody was generated in collaboration with Dr Neil Christensen’s lab. Images of stained monolayers were acquired on a Nikon Eclipse Ti inverted microscope, and fluorescent nuclei were quantified using NIS Elements software.

Samples for western blotting were collected at the time points indicated in RIPA buffer with protease inhibitors and processed as described in the western blotting section. For RT-qPCR array analysis, untransduced or transduced MRC-5 cells were either mock-infected or infected with WT-mCh or UL88-STOP-mCh as described above and harvested 7 days post-infection for RNA isolation as described below.

### THP-1 cell infection by spinoculation

THP-1 (2.5 × 10^5^) cells were cultured in XVIVO medium for 48 hours prior to infection and then infected at 20 MOI. After the addition of virus to the cells, the plate was centrifuged at 1,000 × *g* for 30 min at 22°C. The cells were then incubated at 37°C. 24 hours post-infection, cells were washed twice with 1× PBS by centrifugation at 100 × *g* for 8 minutes. Fresh 10% RPMI media was added to the cells. Cells were incubated at 37°C for an additional 3 days, at which point we assessed that approximately 10% of the cells (2.5 × 10^4^) were infected in each experiment. On day 3, infected THP-1 cells were collected in a tube, centrifuged at 100 g, 8 minutes. Cells were washed with 1× PBS, and the cell pellet was resuspended in fresh 10% RPMI media and added onto a monolayer of HDF that had been seeded 24 hours previously. Each well contained approximately 3 × 10^5^ cells, so the ratio of infected THP-1 cells to HDF cells in the monolayer was approximately 1:12. Cell spread was monitored by imaging every day. Imaging was performed on the Nikon Eclipse Ti inverted confocal system, and images were processed using NIS Elements software to quantify the spread.

### Virus spread assay in transduced MRC-5 cells

MRC-5 cells were transduced with a lentiviral vector or lentivirus expressing MyD88 and selected with puromycin as described above. The selected cells were then infected with the mCherry-tagged TB40/E WT virus at an MOI of 0.05. Cells were monitored for virus spread by imaging on a Nikon Eclipse Ti inverted microscope every three days up to 15 days post-infection. Images were processed using NIS Elements software to quantify the spread by quantifying the fold change in area of fluorescence.

### Spread assay to determine extrinsic and intrinsic factors involved in HCMV spread

First, MRC-5 cells transduced with either vector or MyD88 were infected at an MOI of 0.05 with mCherry-WT TB40/E virus. Supernatants were collected every 3 days until day 15. Supernatants were filtered through a 0.2 µm filter (to remove virus) and frozen at −80°C. Subsequently, another monolayer of MRC-5 cells was infected at an MOI of 0.05 with mCherry-TB40/E WT virus, and the previously filtered supernatants from each time point were added onto the infected cells on every third day by removing the equivalent volume of the filtered supernatant to be added from the well (therefore 1:1 ratio with the media in the well). The spread of the virus was monitored by confocal imaging.

To examine the requirement for biologically active soluble factors in the supernatant in the modulation of HCMV spread, infection was carried out as described above, and supernatants were harvested on day 7. Samples were filtered through a 0.2 µm filter (to remove virus), and part of the samples were boiled at 95°C for 10 min to degrade any soluble factors. The unboiled and boiled supernatants were aliquoted and stored at −80°C until use. Subsequently, another monolayer of MRC-5 cells was infected as above, and previously filtered and either boiled or un-boiled supernatants from each time point were added onto the infected cells on every third day, by removing the equivalent volume of the filtered supernatant to be added from the well (therefore 1:1 ratio with the media in the well). The spread of the virus was monitored by confocal imaging. Imaging was performed on the Nikon confocal microscope system, and images were processed using NIS Elements software to quantify the spread.

### Transfections and immunoprecipitation

For immunoprecipitations from transfected cells, 293 cells were transfected with 1 µg each of the GFP-tagged UL88 Full length, UL88-N (1–180 aa), and UL88-C (181–429 aa) and either the pCDNA3.1 vector or flag-tagged pCDNA3.1-MyD88 expression construct by using the X-treme GENE HP DNA transfection reagent (Roche) according to the manufacturer’s protocol. Total protein was harvested at 24 h post-transfection. All cells were harvested in lysis buffer containing protease inhibitors (1× PBS + 0.2% Triton + 1 mM phenylmethylsulfonyl fluoride (PMSF), 1 mM aprotinin, 0.2 mM Na_3_VO_4_, 1 µg/mL leupeptin). Immunoprecipitations were performed using 500 µg of total protein incubated with FLAG magnetic beads (Sigma-Aldrich M8823) per the manufacturer’s protocol. Briefly, lysates were rotated overnight at 4°C with the beads, washed three times with lysis buffer, and prepared for western blot analysis as described below.

### Western blotting

Total cell lysates were prepared from HCMV-infected cells at an MOI of 3 at 96 hpi/120 hpi or day 12 by harvesting lysates in RIPA buffer (containing 1 mM phenylmethylsulfonyl fluoride (PMSF), 1 mM aprotinin, 0.2 mM Na_3_VO_4_, 1 µg/mL leupeptin), which were then incubated for 10 min on ice. Where applicable, DMSO (Sigma-Aldrich C6295), 50 µM chloroquine (MilliporeSigma), or 10 µM MG132 (ApexBio A2585) was added 12–16 hours before harvest.

The samples were then quantified and boiled with 3× SDS loading dye for 5 min at 95°C. Samples were analyzed by SDS-PAGE and immunoblotting on a polyvinylidene difluoride (PVDF) membrane blocked with 5% milk or 5% bovine serum albumin (BSA) in Tris-buffered saline (0.1% Tween). Primary antibody (overnight at 4°C) and secondary antibody (RT for 1 hour) incubation was done. Blots were developed using the SuperSignal West Pico chemiluminescent substrate (Thermo Scientific) on the ChemiDoc MP Imaging System (Bio-Rad).

### Antibodies

MyD88 (CST cat # 4283), IRF3 (CST cat # 11904), STAT1 (CST cat # 14994), p-STAT1 (BD Biosciences cat # BD612132), p115 (cat # 10090-922), actin (EMD Millipore MAB1501), NF-κB p65 (CST cat # 8242), IRF1 (CST cat # 8478), JAK1 (CST cat # 29261), HA (Abcam ab18181), anti-FLAG (Sigma-Aldrich F1804), EEA1 (Thermo scientific- MA5-14794), and alpha-tubulin (Sigma-Aldrich cat # T9026). Anti-GFP rabbit antibody was a gift from Dr. John Wills. IE-1 mouse antibody was generated in collaboration with Dr. Neil Christensen’s lab. A rabbit antibody against a UL88 peptide was generated by Genscript. The viral anti-pp65 (7B4) was a gift from Dr. David Spector. Horseradish peroxidase-conjugated secondary antibodies for Western blotting detection were purchased from Santa Cruz (goat anti-rabbit) and GE Healthcare (sheep anti-mouse). Antibody dilutions were in accordance with the manufacturer’s instructions. Alexa Fluor, 488-, 568-, and 647-conjugated secondary antibodies (Invitrogen) were used as secondary antibodies for immunofluorescence.

### RNA isolation and RT-qPCR

Total RNA was isolated using RNeasy Mini Kit (cat# 74034) under RNase-free conditions according to the manufacturer’s instructions and precipitated with isopropanol. DNA contaminants were removed by DNase treatment using a DNA-free DNA removal kit (Qiagen cat# 79254). RNA concentrations were determined, and equal amounts were used to generate cDNA using the Invitrogen SuperScript III First-Strand Synthesis System (cat# 18080051) with oligo dT primers according to the manufacturer’s protocol. Equal amounts of cDNA were analyzed by real-time quantitative PCR (qPCR) in triplicate using Power SYBR green master mix (Applied Biosystems) on the Applied Biosystems (ABI) real-time qPCR machine. Primers are as follows:

MyD88 Forward 5′ACCCAGCATTGGTGCCG3′, Reverse 5′GGTTGGTGTAGTCGCAGACA3′;

GAPDH Forward 5′ACCCACTCCTCCACCTTTGAC3′, Reverse 5′CTGTTGCTGTAGCCAAATTCGT3′;

IL1β Forward 5′AAACAGATGAAGTGCTCCTTCCAGG3′, Reverse 5′TGGAGAACACCACTTGTTGCTCCA3′

For qPCR array, RNA isolation and cDNA synthesis were performed with kits from Qiagen (cat# 74034) and RT^2^ First strand kit per manufacturer’s instructions, respectively. Real-time PCR using the RT^2^ Profiler PCR arrays in combination with RT^2^ Syber green master mixes using pathway-specific primer mix (Human Type I Interferon Response, catalog # PBH-016Z; Qiagen) was done for the expression analysis of 84 known ISGs. β-Actin (ACTB) and GAPDH were used as housekeeping controls. Genes with a quantification cycle (Ct) value > 35 were regarded as undetected and assigned a Ct of 35 to calculate fold induction.

### Immunofluorescence staining and imaging

Coverslips with MRC-5 cells-transduced, uninfected, or HCMV-infected cells were fixed in 4% paraformaldehyde for 15 min at room temperature. Cells were blocked in PBS containing 10% human serum, 0.5% Tween-20, and 5% glycine. Triton X-100 (0.1%) was added for permeabilization. Primary and secondary antibodies were diluted in blocking buffer and incubated for one hour each at room temperature. The primary antibody to NF-κB (CST cat# 8242) was used. Alexa Fluor 488, Cy5, or Alexa Fluor 568-conjugated secondary antibodies (Invitrogen) were used as secondary antibodies. Coverslips were mounted with ProLong Diamond antifade containing DAPI (Thermo Scientific). Images were taken on a confocal microscope system (Nikon). Images were processed using NIS Elements software.

### Statistical analysis

Prism GraphPad was used for statistical analysis and generation of graphs. For analysis of the qPCR array, *P*-values were calculated for each gene using a Mann-Whitney signed rank test, and a rank-based volcano plot was created based on the raw *P* values and the fold expression relative to the mean of the controls.

## Data Availability

All primary unanalyzed and unmanipulated data associated with every figure panel are available in Figshare at https://figshare.com/projects/The_HCMV_tegument_protein_UL88_degrades_MyD88_and_reduces_innate_immune_activation/252842.
